# Gzmk^+^ CD8 T cells in inflammatory diseases

**DOI:** 10.3389/fimmu.2025.1661755

**Published:** 2025-08-21

**Authors:** Cui Xin, Peiyun Liu, Qianqian Zhan, Wenqiang Cao

**Affiliations:** ^1^ Department of Criminal Science and Technology, Hunan Police College, Changsha, China; ^2^ Key Laboratory of Major Chronic Diseases of Nervous System of Liaoning Province, Health Sciences Institute of China Medical University, Shenyang, China

**Keywords:** GZMK, CD8, inflammatory disease, inflammaging, aging

## Abstract

T cells are integral to the immune response, with distinct subsets exhibiting specialized functions, a phenomenon well-characterized in helper CD4^+^ T cells. Recent advancements in single-cell RNA sequencing (scRNA-seq) have facilitated the identification of numerous novel CD8 T cell subsets, each characterized by unique functional properties. As cytotoxic T lymphocytes, the primary focus has been on the cytotoxic capabilities and antigen specificity of these subsets. A recently identified subset, Granzyme k (Gzmk)^+^ CD8 T cells, has been closely associated with inflammatory diseases, independent of their cytotoxic function. Unlike other granzymes, granzyme K predominantly induces proinflammatory responses in tissues or cells rather than mediating cytotoxicity. This review synthesizes current evidence regarding the regulation, functional roles, and underlying mechanisms of Gzmk^+^ CD8 T cells in inflammatory conditions. Elucidating these processes may reveal potential therapeutic targets for treating inflammatory diseases.

## Introduction

Inflammatory diseases are mediated by immune cells, which can act in either an autoimmune or non-autoimmune manner. In autoimmune disorders such as rheumatoid arthritis (RA) and multiple sclerosis (MS), CD4 T cells were traditionally considered the primary effectors. However, autoreactive CD8 T cells also play a pathogenic role in several autoimmune diseases by directly damaging self-cells (1). Additionally, CD8 regulatory T cells can negatively impact autoimmune responses by killing autoreactive CD4 T cells (2–4). These functions related to autoimmune activity are associated with cytotoxicity. In non-autoimmune diseases, CD8 T cells are often found in higher concentrations in inflamed tissues, suggesting they likely play a critical role in these conditions as well (5).

Response to different stimulatory milieus, CD4 T cells differentiate into distinct subsets with specialized phenotypes and functions (6). Similarly, CD8 T cells are also categorized into subsets such as Tc1, Tc2 and Tc17, but this classification is controversial (7). Upon stimulation, naïve CD8 T cells differentiate into effector/memory T cells. Effector CD8 T cells can directly kill target cells through interactions involving Fas/Fas ligand, or by secreting perforin and granzymes. Within effector or memory CD8 T cells, several different populations have been identified, including short-lived effector CD8 T cells, long-live memory CD8 T cells (Tm), exhausted CD8 T cells (Tex), memory precursor CD8 T cells (Tmp), central and effector memory CD8 T cells (Tcm and Tem), tissue-resident memory cells (Trm) (8) and terminally differentiated T effector memory CD45RA^+^ (Temra) in human (9). However, these classifications do not entirely capture the specific functions of CD8 T cells in particular diseases. Recent scRNA-seq studies have revealed that Gzmk+ CD8 T cells are enriched in the inflamed tissues of both autoimmune and non-autoimmune diseases (10–16). In this review, we have included all diseases in which the presence of Gzmk^+^ CD8 T cells has been identified using single-cell RNA sequencing (scRNA-seq) and flow cytometry. We will discuss the differentiation, function, and underlying mechanisms of Gzmk^+^ CD8 T cells in the context of inflammatory diseases.

## Identification and generation of Gzmk^+^ CD8 T cells

Conventional subsets such as Tem, Tcm, and Temra do not fully reflect the heterogeneity of CD8 T cells. Advances in single-cell RNA sequencing (scRNA-seq) have enhanced our understanding of CD8 T cell diversity, leading to the identification of several new CD8 T cell subsets in specific diseases or conditions. For instance, Gzmk^+^ CD8 T cells have been identified in aging tissues in mice, exhibiting exhaustion-like phenotypes characterized by the co-expression of TOX, PD1, and other co-inhibitory receptors (17). Further studies have revealed that Gzmk^+^ CD8 T cells also increase in peripheral blood mononuclear cells (PBMCs) from older individuals (17). Aging brings significant changes to CD8 T cells, including an increase in Temra and virtual memory cells (18–20). A key question is whether Gzmk^+^ CD8 T cells represent a specific age-related subset of CD8 T cells. Gzmk^+^ CD8 T cells are primarily found in the Tem and Tcm subsets and are less prevalent in the Temra. Notably, Gzmk^+^ Tem cells are characterized as CD27^+^ CD28^+^ CD57^-^. Transcriptional analysis reveals that Gzmk^+^ CD8 T cells differ from virtual memory cells, which are driven by IL-15 stimulation and do not express CD49d (18). In contrast, Gzmk^+^ CD8 T cells exhibit high CD49d expression (17). Therefore, Gzmk^+^ CD8 T cells should not be classified as typical age-related CD8 T cells. Moreover, it has been observed that CD39^+^ memory CD8 T cells are elevated in older individuals (21), and CD39 expression is also a hallmark of cell exhaustion (22–24), raising the question of whether Gzmk and CD39 co-express in the same CD8 T cell population. Although Gzmk and granzyme b (Gzmb) are both granzymes, their expression profiles differ significantly. Cytometry and scRNA-seq analyses have demonstrated that Gzmk and Gzmb express mutually within the Tem subset (17). In summary, Gzmk^+^ CD8 T cells are a distinct subset associated with aging, highlighting the complexity of CD8 T cell heterogeneity.

Gzmk^+^ CD8 T cells were initially identified as conserved age-associated T cells (Taa). Still, they have also been found to be enriched in inflamed tissues and circulation during many inflammation-related diseases (10, 12–16, 25–28). This suggests that Gzmk^+^ CD8 T cells play a crucial role in these diseases, in addition to their involvement in age-related immune responses. However, the reasons for Gzmk^+^ CD8 T cells accumulating in aged or inflamed tissues remain unclear. Mogilenko et al. revealed that the old environment drives the generation of Gzmk^+^ CD8 T cells (17), indicating that shared factors between aged and inflamed tissue are responsible for their differentiation. Single-cell ATAC-seq analysis suggests that the transcriptional activity of Eomes is elevated, which may be involved in the differentiation of Gzmk^+^ CD8 T cells (17). Eomes has been shown to promote CD8 T cells exhaustion and directly regulate exhaustion-associated genes (29, 30), raising a question of whether Eomes is involved in the regulation of Gzmk. Indeed, Eomes was reported to bind to the promoter of *Gzmk* and induce its expression in CD4 T cells (31, 32). This leads to the speculation that factors within inflamed and aged tissues trigger Eomes expression, which in turn causes the generation of Gzmk+ CD8 T cells, warranting future investigation.

## The function and regulation of Gzmk in CD8 T cells

Granzymes are primarily recognized as factors that induce cell death in target cells. For instance, Gzmb enters the target cell and cleaves caspases 3 and 7 to trigger apoptosis (33). The delivery of Gzmb relies mainly on the formation of pores in the cell membrane mediated by perforin (34). Early studies on Gzmk also focused on its cytotoxicity, demonstrating that Gzmk can induce caspase-independent apoptosis by cleaving various substrates, including SET, Ape1, HMG2, Bid, VCP and P53 (35–39). However, Gzmk^+^ CD8 T cells lack perforin (10, 14), and extracellular Gzmk is present in specific conditions (40), suggesting Gzmk has additional functions beyond its intracellular cytotoxicity. Increasing evidence indicates that Gzmk plays a proinflammatory role by cleaving extracellular substrates such as PAR1, lipopolysaccharides (LPS) and complement proteins (12, 13, 40–44).

Gzmb and perforin in CD8 T cells are synthesized and released upon T-cell receptor (TCR) activation and the formation of the immunological synapse (45, 46). Interestingly, Gzmk is constitutively synthesized and released by CD8 T cells, even in the absence of TCR stimulation. In fact, TCR activation inhibits Gzmk expression in human CD8 T cells (14, 41). Notably, co-inhibitory receptors, such as PD-1, LAG3 and CTLA4, are elevated at both human and murine Gzmk^+^ CD8 T cells (17), which likely play a role in regulating Gzmk expression by counteracting the inhibition mediated by TCR signaling. Furthermore, aged CD8 T cells tend to acquire natural killer (NK)-related phenotypes and downregulate TCR-related molecules (47). This results in reduced TCR signaling transduction and makes aged CD8 T cells more sensitive to cytokine stimulation. IL-2/IL-12 strongly induces Gzmk expression and proliferation of Gzmk^+^ CD8 T cells (14), while IL-15 is also capable of triggering Gzmk expression in CD8 T cells (28).

There is a complex interaction between cytokines and TCR signals that determines the differentiation fate of CD8 T cells. A recent study has shown that TCR signaling inhibits the IL-15-induced the upregulation of NK-related genes through NFATc1 (48). While IL-15 activates AP-1 to induce NKG2D expression, the binding of NFATc1 to AP-1 constrains this effect. In contrast to wild-type NFATc1, overexpression of a mutant form of NFATc1 that cannot bind to AP-1 does not abrogate IL-15-induced NKGD2 upregulation (48). Interestingly, expression of a version of NFAT1 that is incapable of interacting with AP-1 induced exhaustion of CD8 T cells characterized by upregulation of inhibitory receptors and the related transcription factors including BLIMP1, ZEB1 and TOX (49). TOX is highly expressed in Gzmk^+^ CD8 T cells, but it remains unclear whether TOX regulates the function and differentiation of Gzmk^+^ T cells through integrating cytokines and TCR signaling.

ATAC-seq and RNA-seq analysis indicated that Eomes is likely an essential transcription factor in human and murine Gzmk^+^ CD8 T cells. Tem-k (Gzmk^+^ Tem) and Tem-b (Gzmb^+^ Tem) cells were enriched for Eomes and T-bet motif, respectively (17). Moreover, Eomes plays a critical role in the effects of IL-15 and has been reported to regulate Gzmk expression directly in CD4 T cells (50). These suggest that the differentiation of Tem-k and Tem-b may rely on a balance between T-bet and Eomes-driven transcriptional regulation, similar to the differentiation of effector and exhausted CD8 T cells (51, 52). Notably, the ratio of T-bet/Eomes has been reported to be influenced by the strength of TCR signaling, with low-affinity peptides favoring a lower ratio of T-bet/Eomes (53). Further investigation is warranted to determine whether TCR or/and cytokines signaling regulates the differentiation of Gzmk^+^ CD8 T cells through the transcriptional activity of T-bet and Eomes. Additionally, in the absence of TCR stimulation, IL-15 promotes the generation of virtual memory cells, which also dependents on Eomes (18, 54). Future research should explore how Eomes responds to environmental stimuli and controls the differentiation of virtual memory, exhausted and Gzmk^+^ CD8 T cells.

## Gzmk^+^ CD8 T cells in autoimmune diseases

The role of CD8 T cells in autoimmune diseases has been extensively studied, which depends on their cytotoxicity towards self-cells or autoreactive CD4 cells (1–4). Recent researches have revealed that Gzmk^+^ CD8 T cells are enriched in tissues of rheumatoid arthritis (RA) and psoriasis, where they contribute to inflammation and disease progression by activating the complement system (10, 41). Notably, Gzmk has been identified in areas abundant with C3b and C5a. Experiments have shown that deficiency of Gzmk in mice leads to reduction in the severity of RA, imiquimod-induce dermatitis, and C3d deposition (41). Given that the crucial function of complements in autoimmune diseases (55), It is likely that Gzmk^+^ CD8 T cells are involved in autoimmune diseases via activating complement system. Beyond RA and psoriasis, Gzmk^+^ CD8 T cells have also been found to be enriched in at tissues of systemic lupus erythematosus (SLE) (56, 57), Sjögren’s disease (28), uveitis (58) and IgG4-related disease (59). The function of complements in autoimmune diseases is complex, exhibiting pathogenic or protective roles (55). For instance, deficiencies of C1q and C4, can exacerbate the severity of SLE, whereas a lack of C3 may alleviate it (55). Gzmk is reported to cleave C2 and C4, resulting in the formation of C3 convertase, which generates C3a and triggers a pro-inflammatory reponse (12, 41). This mechanism likely contributes to the progression of RA, psoriasis, SLE and uveitis (60). In cases of Sjögren’s disease and IgG4-related diseases, a higher frequency of hypocomplementemia is observed which is linked to the clinical phenotype (61, 62).Therefore, further investigation is needed to determine whether Gzmk^+^ CD8 T cells contribute to Sjögren’s disease and IgG4-related diseases through the activation complements.

Apart from its roles in cleaving complements, Gzmk has also been reported to cleave protease-activated receptor 1 (PAR1), which significant in inflammation, including autoimmune dieases (63). For example, *PAR1^-/-^* mice exhibited less severity of arthritis induced by antigen (AIA) (64), which reflects the role of Gzmk in AIA (41). However, the deficiency of PAR1 exacerbated the collagen-induced arthritis (CIA) (65). This complexity is further highlighted by the fact that PAR1 serves different functions in various cells types involved in inflammatory bowel disease (IBD) (66), another autoimmune condition. Notably, Gzmk^+^ CD8 T cells have also been identified in the lamina propria and intraepithelial of patients with ileal Crohn’s disease (67). Hence, the roles of Gzmk-mediated PAR1 cleavage and activation at RA and IBD warrant further investigation.

Lipopolysaccharide (LPS) is another extracellular substrate of Gzmk (42), which strongly stimulates the innate immune response. This stimulation can exacerbate autoimmune conditions, such as SLE (68), autoimmune uveitis (69), IBD (70) and RA (71). Gzmk binds to LPS and pulls it out of its micelle conformation, enhancing the stimulation of immune cells by LPS (42). Therefore, in the context of bacterial infection or LPS leakage from damaged intestinal epithelium, Gzmk may worsen autoimmune diseases by amplifying the LPS-induced pro-inflammatory response.

Unlike Gzmk^+^ CD8 T cells, Gzmb^+^ CD8 T cells play a significant role in autoimmune diseases through their cytotoxicity (72). On one hand, auto-reactive Gzmb^+^ CD8 T cells kill target cells and cleave proteins to release autoantigens, which in turn promote the generation of autoantibodies (73, 74). On the other hand, Ly49^+^/KIR+ Gzmb^+^ CD8 T cells help prevent an excessive autoimmune response by targeting and eliminating autoreactive CD4 T cells (2–4, 75–78). This adds an additional layer of regulation by CD8 T cells to the complexity of autoimmune disease.

## Gzmk^+^ CD8 T cells in airway inflammatory diseases

Clonally expanded Gzmk^+^ CD8 T cells are enriched in nasal polyps from chronic rhinosinusitis patients (12, 13), which are associated with type 2 immune cells (79). Research by Guo et al. demonstrated that Gzmk^+^ CD8 T cells interact with fibroblasts via the CXCR4-CXCL12 axis, inducing fibroblasts to release neutrophil chemoattractants. This exacerbated the airway inflammation and resistance to medical intervention in chronic rhinosinusitis with nasal polyps (CRSwNP) (13, 80, 81), where Gzmk^+^ CD8 T cells likely play a crucial role. TCR analysis revealed that Gzmk^+^ CD8 T cells show a selective expansion of clones that recognize Epstein-Barr virus (EBV) (13). This indicates that Gzmk^+^ CD8 T cells are involved in airway inflammation by a bystander way, which aligns with the observation that Gzmk can be released by CD8 T cells independently of TCR stimulation (14, 41). Deletion of Gzmk in T cells resulted in reduced numbers of neutrophils, eosinophils, CD4 T cells, dendritic cells and macrophages in bronchoalveolar lavage fluid (BALF) in mice with asthma (12), This suggests that Gzmk is essential not only for the recruitment of neutrophil, but also for the recruitment of type 2 immune cells, likely due to chemokines and C3a produced locally (12). Gzmk^+^ CD8 T cells cannot promote eosinophil infiltration into the airway in *C3*-deficienct mice (12), suggesting the recruitment depends on C3 cleavage. Differently, the recruitment of neutrophils mainly depends on the interaction between Gzmk^+^ CD8 T cells and fibroblasts. This interaction has also been observed in the context of aging via the FN1-CD49d pathway (17). This leads to a hypothesis that fibroblasts from aged or inflamed tissues attract and retain Gzmk^+^ CD8 T cells, which in turn stimulate fibroblasts to produce cytokines and chemokines and generate activated C3, thereby enhancing inflammation.

In addition to directly stimulating to fibroblasts, Gzmk can cleave complements produced by fibroblasts to generate C3a, which stimulates inflammasome activation and cytokines production at fibroblasts (41, 82). The specific mechanisms by which Gzmk induces an inflammatory response in fibroblasts remain unclear, but studies involving other cell types may offer informative insights. Gzmk has been shown to trigger endothelial activation and promote the production of CCl2 and IL-6 (43). Since fibroblasts also express PAR1 and its activation induces CCL2 expression in these cells (83), it is likely that Gzmk activates fibroblasts through cleaving PAR1 as well. In summary, Gzmk^+^ CD8 T cells, whether generated locally or immigrated, drive airway inflammation by directly activating fibroblasts or by producing activated C3.

## Gzmk^+^ CD8 T cells in age-related diseases

Gzmk^+^ CD8 T cells were initially recognized as conserved age-related T cells during aging. This suggests that Gzmk^+^ CD8 T cells may increase the susceptibility of older individuals to age-related diseases, such as neurodegenerative disorders and cardiovascular disease. We summarize the current evidence regarding Gzmk^+^ CD8 T cells in the context of age-related diseases in this section.

Alzheimer’s disease (AD) is an age-related neurodegenerative disorder characterized by neuroinflammation, and is considered an inflammatory disease (84). Recent studies indicate that CD8 T cells play a significant role in AD (85–89), with both pathogenic and protective effects reported. Therefore, it is essential to identify specific subsets and closely investigate their function in AD. Notably, Gzmk^+^ CD8 T cells have recently been reported to be enriched in the brains of tauopathy mice and deposited onto microgila (16). Depletion of CD8 T cells promoted the spread of phosphorylated tau (pTau) throughout the central nervous system (CNS), suggesting that Gzmk^+^ CD8 T cells may slow the tauopathy progression (16). In a different study, infiltration of CD8 T cells mediated by microglia was found to promote the tauopathy in *APOE4* knock-in/Tau P301S mice (TE4) (89). Although the authors did not examine the expression of Gzmk or the Gzmk^+^ CD8 T cells, they identified a TOX^+^ PDCD1^+^ subset that was decreased in TE4 mice, which is likely related to Gzmk^+^ CD8 T cells (17).

The presence of the blood-brain barrier (BBB) significantly influences the profile of T cells in the brain. Gzmk^+^ CD8 T cells express higher CD49d, which forms very late antigen-4 (VLA-4). VLA-4 interacts with VCAM-1 at brain endothelial cells (BEC) and facilitates the infiltration of CD8 T cells into the brain, which has been reported to be upregulated at the BECs of AD mice (90). Additionally, higher levels of soluble VCAM-1 in plasma from AD patients correlate with advanced dementia (91). Therefore, Gzmk^+^ CD8 T cells are selectively infiltrating into the brains of AD patients via the VLA-4-VCAM-1 pathway. Furthermore, Gzmk can stimulate endothelial cells to upregulate VCAM-1 (43). Consistently, CCR5^+^ GZMK^+^ CD8 T cells have been observed entering the CNS parenchyma of multiple sclerosis patients via GZMK-mediated transendothelial diapedesis (92). This suggests that Gzmk^+^ CD8 T cells have a greater advantage in translocating to the brains of AD patients. The evidence about Gzmk+ CD8 T cells in AD is indirect and limited; their role in AD is uncertain. Since complements and PAR1 in brains, known substrates of Gzmk, have important roles in AD (93, 94), and the close interaction between Gzmk+ CD8 T cells and microglia, it is particularly interesting to examine the function of Gzmk+ CD8 T cells in AD using specific inhibitors or knock-out mouse models.

Sepsis results in increased mortality among the elderly (95, 96), which is linked to a dysfunctional innate immune response. Pro-inflammatory agents, such as LPS from the intestinal microbiome, leak from damaged intestines during aging, which is associated with inflammaging (97, 98). Given that Gzmk can enhance the LPS-induced response and trigger the production of pro-inflammatory cytokines in human monocytes and mouse macrophages respectively (42, 99), the presence of increased Gzmk^+^ CD8 T cells may make old individuals more susceptible to sepsis. Therefore, targeting Gzmk^+^ CD8 T cells presents a potential therapeutic opportunity for addressing sepsis in the elderly population.

Atherosclerosis and its associated cardiovascular complications are major causes of death worldwide. Aging is a significant risk factor for atherosclerosis, which is classified as an age-related inflammatory disease (100). However, the mechanisms by which aging promotes atherosclerosis remain unclear. Research has shown that Gzmk^+^ CD8 T cells are enriched in aortas from old *Ldlr-/-* mice, which develop atherosclerosis on a regular chow diet (CD). The presence of GZMK^+^ CD8 T cells has also been confirmed in human atherosclerostic plaque (15, 101). This suggests that Gzmk^+^ CD8 T cells likely play a role in the development of atherosclerosis in the context of aging. Notably, PAR1 plays a crucial role in atherosclerosis due to its pleiotropic effects on endothelial cells and macrophages (102). Vorapaxar, a selective PAR1 antagonist, has been shown to reduce atherogenesis, plaque vulnerability and complications (102). This suggests that Gzmk from Gzmk+ CD8 T cells in older individuals may play a crucial role as an activator of PAR1 in the context of atherosclerosis. Additionally, complements also play vital roles in atherosclerosis (103), Gzmk may exacerbate the development of atherosclerosis by activating both complements and PAR1, which warrants further investigation.

## Concluding remarks

**Figure 1:**
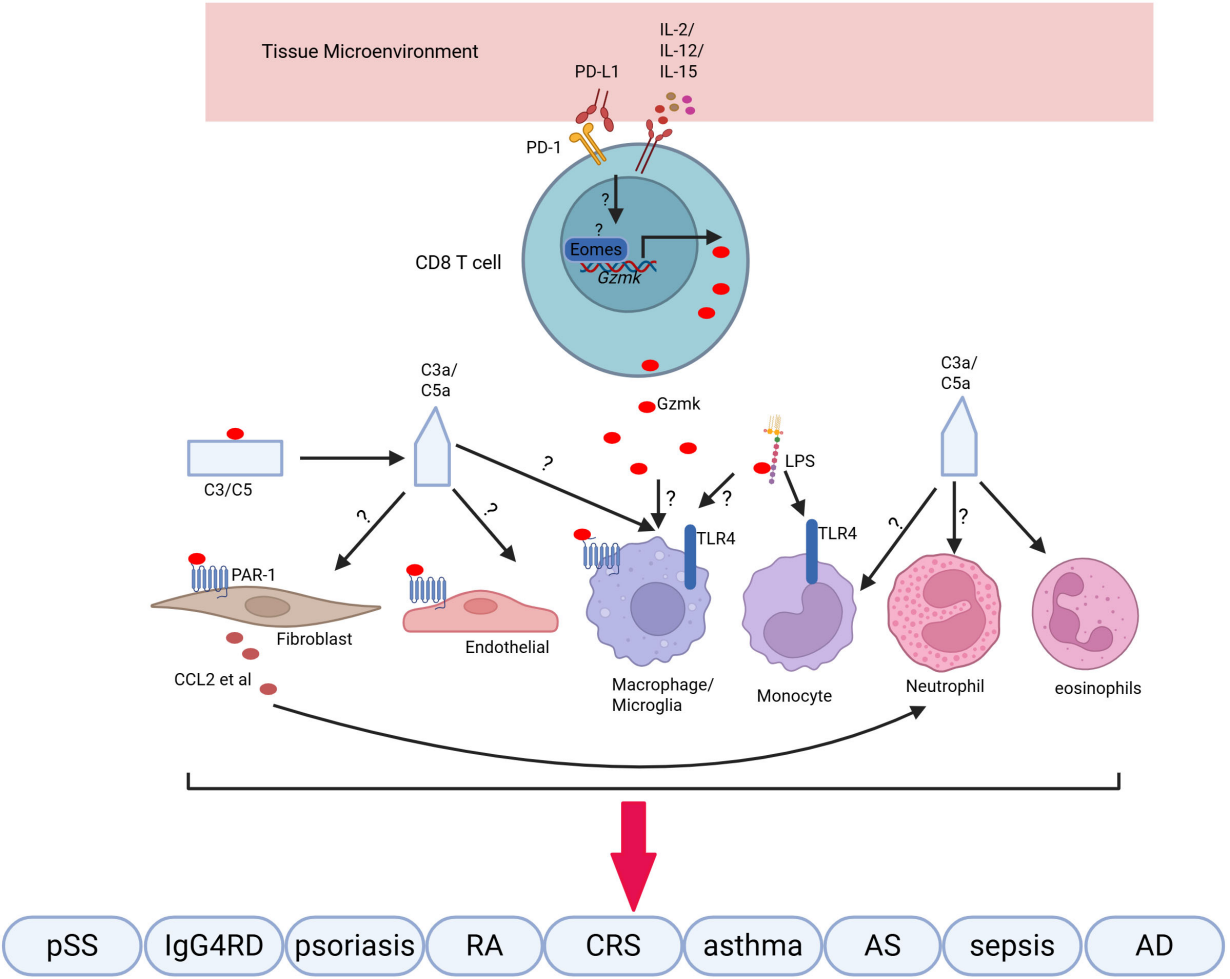
Gzmk^+^ CD8 T cells synthesize and release Gzmk in response to cytokines. Extracellular Gzmk cleaves and activates PAR-1 on various cells, enhancing the inflammatory response by promoting the production of cytokines and chemokines, which recruit immune cells. Additionally, Gzmk cleaves complement proteins to generate C3a and C5a, further amplifying the inflammatory response. Finally, Gzmk binds to LPS, extracting them from their micelles and potentiating the LPS-induced pro-inflammatory response. These pro-inflammatory responses contribute to inflammation in various inflammatory diseases.

Gzmk^+^ CD8 T cells are a population of age-related T cells that accumulate in tissues as we age. This population is enriched in inflamed tissues associated with various inflammatory conditions. Gzmk plays a pro-inflammatory role by cleaving extracellular substrates, including PAR1, complement proteins, and LPS, rather than exhibiting its cytotoxic effects. This may contribute to the phenomenon of inflammaging (**Figure 1**). While numerous studies show a correlation between Gzmk^+^ CD8 T cells and various inflammatory diseases, the underlying mechanisms remain limited and unclear. In addition to the known substrates, Gzmk may cleave new substrates, potentially playing unknown roles under specific conditions. Further investigations are needed to examine the factors that drive the differentiation of Gzmk^+^ CD8 T cells and the signaling pathways that regulate Gzmk expression. This research will enhance our understanding of the role of Gzmk^+^ CD8 T cells. Overall, Gzmk^+^ CD8 T cells and their substrates represent promising targets for addressing various inflammatory disorders, particularly within the context of aging.

## Future direction

There is mounting evidence that Gzmk^+^ CD8 T cells may play a significant role in various inflammatory conditions by generating specific tissue-resident and/or circulating T cells. However, the origin, differentiation, and distribution profile of Gzmk+ CD8 T cells during aging and specific inflammatory disorders remain unclear. Future investigations should examine the common and tissue-type-specific factors responsible for generating Gzmk^+^ CD8 T cells, as well as how these factors contribute to their circulation. This research could provide potential targets for controlling Gzmk^+^ CD8 T cells. Moreover, Gzmk is the main effector of Gzmk^+^ CD8 T cells, depending on the enzymatic activity of Gzmk. Therefore, natural or small-molecule compounds that can inhibit the catalytic activity of Gzmk present a promising direction for combating inflammaging and other inflammatory conditions.
